# Diagnosis of Schizophrenia Using EEG Sensor Data: A Novel Approach with Automated Log Energy-Based Empirical Wavelet Reconstruction and Cepstral Features

**DOI:** 10.3390/s24206508

**Published:** 2024-10-10

**Authors:** Sumair Aziz, Muhammad Umar Khan, Khushbakht Iqtidar, Raul Fernandez-Rojas

**Affiliations:** 1 Human-Centred Technology Research Centre, Faculty of Science and Technology, University of Canberra, Canberra, ACT 2617, Australia; sumair.aziz@canberra.edu.au (S.A.); raul.fernandezrojas@canberra.edu.au (R.F.-R.); 2 Department of Computer and Software Engineering, National University of Sciences and Technology, Islamabad 44000, ICT, Pakistan; khushbakht.iqtidar18@ce.ceme.edu.pk

**Keywords:** schizophrenia, electroencephalography (EEG), cepstral features, automated log energy-based empirical wavelet reconstruction (ALEEWR), feature extraction, machine learning

## Abstract

Schizophrenia (SZ) is a severe mental disorder characterised by disruptions in cognition, behaviour, and perception, significantly impacting an individual’s life. Traditional SZ diagnosis methods are labour-intensive and prone to errors. This study presents an innovative automated approach for detecting SZ acquired through electroencephalogram (EEG) sensor signals, aiming to improve diagnostic efficiency and accuracy. We utilised Fast Independent Component Analysis to remove artefacts from raw EEG sensor data. A novel Automated Log Energy-based Empirical Wavelet Reconstruction (ALEEWR) technique was introduced to reconstruct decomposed modes based on their variability, ensuring effective extraction of meaningful EEG signatures. Cepstral-based features—cepstral activity, cepstral mobility, and cepstral complexity—were used to capture the power, rate of change, and irregularity of the cepstrum of preprocessed EEG signals. ANOVA-based feature selection was applied to refine these features before classification using the K-Nearest Neighbour (KNN) algorithm. Our approach achieved an exceptional accuracy of 99.4%, significantly surpassing previous methods. The proposed ALEEWR and cepstral analysis demonstrated high precision, sensitivity, and specificity in the automated diagnosis of schizophrenia. This study introduces a highly accurate and efficient method for SZ detection using EEG technology. The proposed techniques offer significant improvements in diagnostic accuracy, with potential implications for enhancing SZ diagnosis and patient care through automated systems.

## 1. Introduction

Schizophrenia is a chronic mental disorder where the human mind becomes disconnected from the real world. The condition is specified by relapsing episodes of psychosis, where the symptoms include hallucination, delusion, and paranoia. The mental disorder influences the thinking ability and general behaviour of a person, hence, his or her personal, family, social, and professional functioning is highly affected [1,2]. Worldwide, every 1 in 300 people (0.32%) is affected by this disease. Roughly 24 million people are affected by this disorder. The World Health Organisation (WHO) declares that schizophrenia is curable with an early diagnosis. With early detection, the patient can be given appropriate health care assistance [3]. Early diagnosis can help in curing or limiting the effects of schizophrenia on a person. No specific clinical test is available for the detection of schizophrenia. Mostly, the diagnosis is made through long interviews with a clinical psychiatrist. No definitive biological sample analysis technique can assure the diagnosis of the disorder [4]. A systematic review is given by Davison et al. [5], where they have enlisted the possibility of diagnosis through metabolomics in the discovery of disease biomarkers. Researchers have been trying to devise non-invasive techniques to diagnose the problem. These techniques include imaging and signal processing methods such as Magnetic Resonance Imaging (MRI) and Computed Tomography (CT) scans. The high costs of these tests are not affordable for everyone. Therefore, signalling techniques are gaining popularity. The human brain activity and functionality can be examined through electroencephalogram (EEG) signals acquired with appropriate electrode placement. An EEG is recorded by placing the electrodes at predefined positions on the scalp. EEG has been used in the detection of multiple brain disorders such as insomnia, dementia, epilepsy, schizophrenia, etc. [6].

EEG signals have been actively studied for schizophrenia detection through the use of machine learning techniques. For instance, Weikoh et al. [7] analysed 1142 EEG signals over 25 s, converting these into images using a spectrogram and extracting local configuration pattern features. This approach achieved a high accuracy of 97.20% using a KNN classifier. Ahmad et al. [8] segmented EEG signals into five standard frequency bands to classify schizophrenia, utilising these segments as features in a support vector machine (SVM) that attained an 89.21% accuracy. Sima et al. [9] transformed sensor-level EEGs to source level, analysing phase lag via a functional connectivity network and employing logistic regression on theta band features from brain-complex network analysis to achieve 97% accuracy. Talha et al. [10] selected electrodes with high signal-to-noise ratios from raw EEG signals, computed linear and non-linear features, and obtained a 93% classification accuracy for schizophrenia. Schizophrenia detection has also been explored using graph theory-based network connectivity analysis, with a study [11] utilising EEG data from 39 healthy and 45 schizophrenic subjects, achieving an 82.3% accuracy with a Random Forest (RF) classifier. Zhang et al. applied machine learning classifiers to event-related potential (ERP) features from EEG signals [12,13]. They achieved classification accuracies up to 98.5% using RF and Artificial Neural Networks (ANNs), incorporating five temporal and two demographic features in the model.

Empirical Mode Decomposition (EMD) is a highly effective method for analysing non-linear and non-stationary signals such as EEG recordings [14,15]. EMD iteratively decomposes a signal into a set of intrinsic mode functions (IMFs) that represent simple oscillatory modes. EMD was employed to decompose the EEG signal into IMFs [14]. Their proposed model extracted entropy features and used a Support Vector Machine classifier to achieve a 0.98 AUC value for the detection of SZ. Siuly et al. [15] applied EMD to decompose EEG signals into IMFs, followed by feature extraction. In the classification step, the ensemble bagged tree offered an overall accuracy of 89.5% using IMF2. Jahmunah et al. proposed an automated diagnosis tool based on non-linear features using a 19-channel EEG for the screening of schizophrenia [16] and obtained an accuracy of 92.91%. However, while EMD is effective, it can suffer from mode mixing and relies heavily on the selection of IMFs.

Deep learning (DL) techniques have also been implemented for the study of schizophrenia. For example, Oh et al. proposed an eleven-layered Convolutional Neural Network (CNN) architecture for schizophrenia recognition using EEG signals [17]; the deep learning model yielded an overall recognition accuracy of 98% for non-subject-based analysis. Singh et al. proposed a spectral features-based CNN architecture for the accurate prediction of schizophrenia using EEG signals [18]; they reported accuracies of 94% and 98.5% classification accuracies for two EEG datasets. Chandran et al. proposed schizophrenia classification using a four-layered long short-term memory (LSTM) network, a type of artificial recurrent neural network [19]; approximate entropy, Katz fractal dimension, and variance features were computed from a 19-channel EEG and passed to LSTM for prediction of disorder. Authors in [20] developed a framework by combining a CNN and logistic regression for the diagnosis of schizophrenia using only three channels of EEG; they reported accuracies of 90% and 98% for the subject-based and non-subject-based experimentation, respectively. In another study, Lillo et al. [4] analysed EEG data from fourteen patients using a high-pass filter and microstate transformation, achieving 93% diagnostic accuracy with a CNN. Wu et al. [21] applied a recurrent auto-encoder model, achieving a classification accuracy of 81.81%. Phang et al. [22] proposed a framework using various time and frequency domain features with a CNN, reaching 91.69% accuracy. In another study, Phang et al. [23] used directed connectivity and complex network measures, achieving an overall accuracy of 95% with Deep Neural Networks.

Existing EEG-based diagnostic methods for schizophrenia face several limitations. Time- and frequency-based methods require careful consideration and may not fully capture the complex, non-linear characteristics of EEG signals. EMD-based methods, while adaptive, can suffer from issues such as mode mixing, where different scales of data are mixed within a single IMF, complicating the analysis and potentially reducing diagnostic accuracy. Deep learning approaches, despite their capability to automate feature extraction, heavily rely on large datasets and substantial computational resources, which limits their practicality in medical settings. These models typically utilise all available EEG channels, increasing computational burdens and the risk of overfitting. Therefore, our proposed computer-aided diagnosis system addresses these limitations by employing Automated Log Energy-based Empirical Wavelet Reconstruction (ALEEWR) for automated noise reduction and selective signal reconstruction. This approach enhances signal processing efficiency and accuracy by proposing cepstral domain features that demonstrate high discriminative power. Unlike traditional methods, our system utilises only ten features and identifies important EEG channels, significantly reducing computational complexity. This targeted and efficient approach not only lowers computational demands, making the system suitable for real-time applications in battery-powered devices, but also achieves superior diagnostic performance. The key contributions of our work are listed below:

1. We propose the Automated Log Energy-based Empirical Wavelet Reconstruction (ALEEWR) for noise reduction in EEG signals.
2. We introduce a novel feature set derived from three cepstral parameters that, when used in our proposed computer-aided diagnosis system, outperforms state-of-the-art methods by classifying schizophrenia and healthy patients through EEG signals using only ten features.
3. This study also identifies crucial EEG channels (e.g., possible biomarkers) that contain distinct information pertinent to schizophrenia. By pinpointing key features and channels, we reduce the computational complexity and enhance the feasibility of continuous monitoring in battery-powered embedded systems.

The structure of this article is organised as follows: Section 2 provides a detailed description of the EEG dataset and outlines the proposed framework for diagnosing schizophrenia, emphasising innovative signal processing and feature extraction techniques. In Section 3, we present the experimental results of our method. Section 4 delivers an in-depth analysis of our approach and compares it with existing studies. Finally, Section 5 summarises the key findings and concludes the article.

## 2. Materials and Methods

### 2.1. Overview

**Figure 1:**
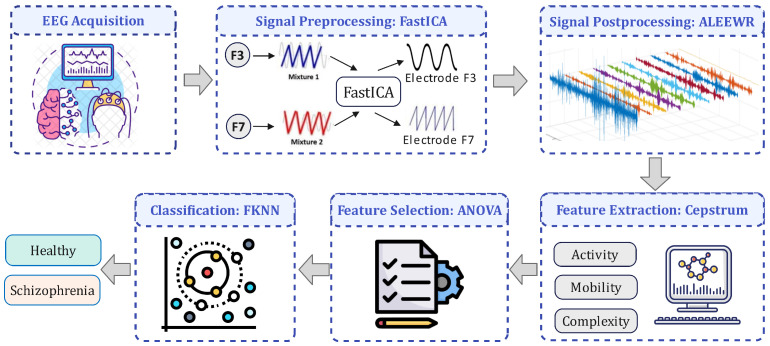
Framework of the proposed computer-aided diagnosis system of schizophrenia.

Our proposed diagnostic framework for schizophrenia utilises advanced signal processing and machine learning techniques to enhance accuracy with EEG data. Figure 1 presents the proposed framework for computer-aided diagnosis of schizophrenia. In the first step, EEG signals are cleaned using FastICA to remove artefacts and isolate independent components. These signals are then processed through Automated Log Energy-based Empirical Wavelet Reconstruction (ALEEWR) to highlight EEG signatures. Cepstral features are extracted from the reconstructed signal, and ANOVA-based feature selection refines these features to those most indicative of schizophrenia. The final step involves classification using a Fine KNN algorithm, effectively distinguishing between healthy individuals and patients with schizophrenia based on EEG data.

### 2.2. EEG Dataset

**Figure 2:**
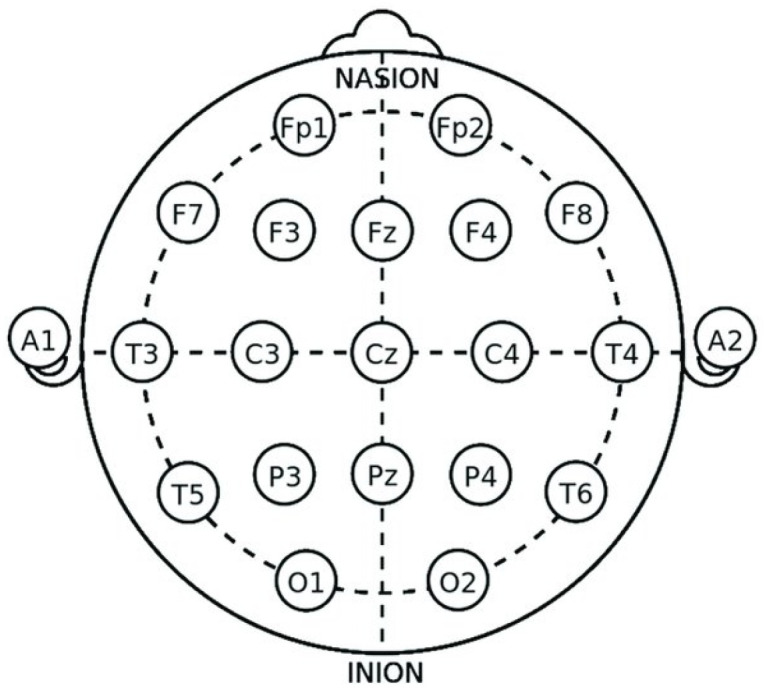
Standard placement of electrodes for EEG acquisition.

In this study, a publicly available dataset of EEGs was employed for the evaluation of the proposed methodology for the detection of schizophrenia (SZ) [24]. The data were acquired at the Institute of Psychiatry and Neurology in Warsaw, Poland. The EEG data contain 19 channels (Fp1, Fp2, F7, F3, Fz, F4, F8, T3, C3, Cz, C4, T4, T5, P3, Pz, P4, T6, O1, O2) obtained at a sampling rate of 250 Hz using standard 10-20 EEG system (Figure 2).

**Figure 3:**
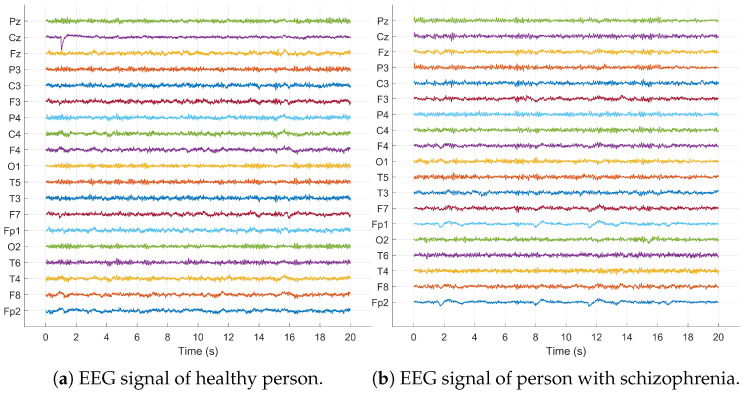
Raw EEG signals of healthy and schizophrenic subjects.

This EEG dataset includes recordings from 14 patients diagnosed with schizophrenia (SZ), consisting of seven females (average age: 28.3±4.1 years) and seven males (average age: 27.9±3.3 years). Additionally, EEG data were gathered from 14 healthy controls, split evenly by gender with seven females (mean age: 28.7±3.4 years) and seven males (average age: 26.8±2.9 years. Recordings were conducted over fifteen minutes while subjects rested with their eyes closed. The signals were then segmented into 20 s intervals, with each segment containing 5000×19 data points. Figure 3 displays a segment of the raw signals from both healthy individuals and those with schizophrenia.

### 2.3. Preprocessing: FastICA

To address the challenges of artefact removal and the separation of EEG channels that are independent of each other, we employed Independent Component Analysis (ICA) [25,26]. ICA is pivotal in enhancing the quality of EEG data by isolating components associated with noise and interference from those reflecting genuine brain activity. ICA endeavours independence by transforming feature space linearly into a new feature space such that each component in the new feature space is mutually independent. Nonetheless, the mutual information between the original and transformed feature space is kept as high as possible. Consider *y* is an input matrix with a dimension of [m×n] where *m* is the total number of samples and *n* is the number of variables. The ICA model is mathematically given as:

(1) z∈R

(2) y=Az

where *A* is the mixing matrix and *z* represents the data from individual sources that indicate the independent components. It is assumed that the data are linearly combined (non-Gaussian data distribution) from individual sources. To reconstruct the independent signal, an unmixing matrix *W* is constructed, which is an inverse of the mixing matrix *A*.

Therefore,

(3) z=Wy

where

(4) $$W=A^{-1}$$

**Figure 4:**
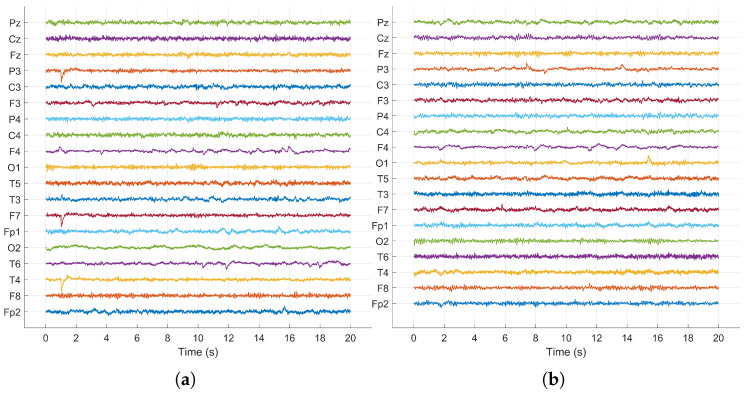
FastICA- based preprocessing of EEG signals. (**a**) FastICA based preprocessing for healthy person. (**b**) FastICA-based preprocessing for person with schizophrenia.

Among the various ICA algorithms available, we opted for FastICA [27,28,29] due to its computational efficiency and robust performance in dealing with biomedical signal processing. The use of FastICA allowed us to effectively clean the EEG data (Figure 4), preparing them for further analysis and ensuring that our feature extraction methods could operate on the most relevant and least contaminated signals.

### 2.4. EEG Postprocessing: Automated Log Energy-Based Empirical Wavelet Reconstruction (ALEEWR)

The field of EEG signal analysis has seen significant advances in preprocessing and feature extraction techniques, with popular methods including Empirical Mode Decomposition (EMD) [15,30], Variational Mode Decomposition (VMD) [31,32,33], and Wavelet Transform (WT) [34,35]. Each of these methods, however, has its specific limitations: EMD faces mode selection issues and mode mixing, VMD suffers from over-decomposition, and WT, despite better energy preservation compared to EMD and VMD, is hindered by the non-adaptive nature of the basis functions.

To address these shortcomings, the Empirical Wavelet Transform (EWT) [36,37,38] emerges as an adaptive method that effectively preserves energy during decomposition. However, EWT itself faces challenges, particularly in deciding which decomposed modes should be selected for reconstructing a preprocessed signal. This selection is crucial as it influences the quality and effectiveness of the subsequent signal analysis, particularly when handling the inherently weak signals typical of human brain EEG data.

**Figure 5:**
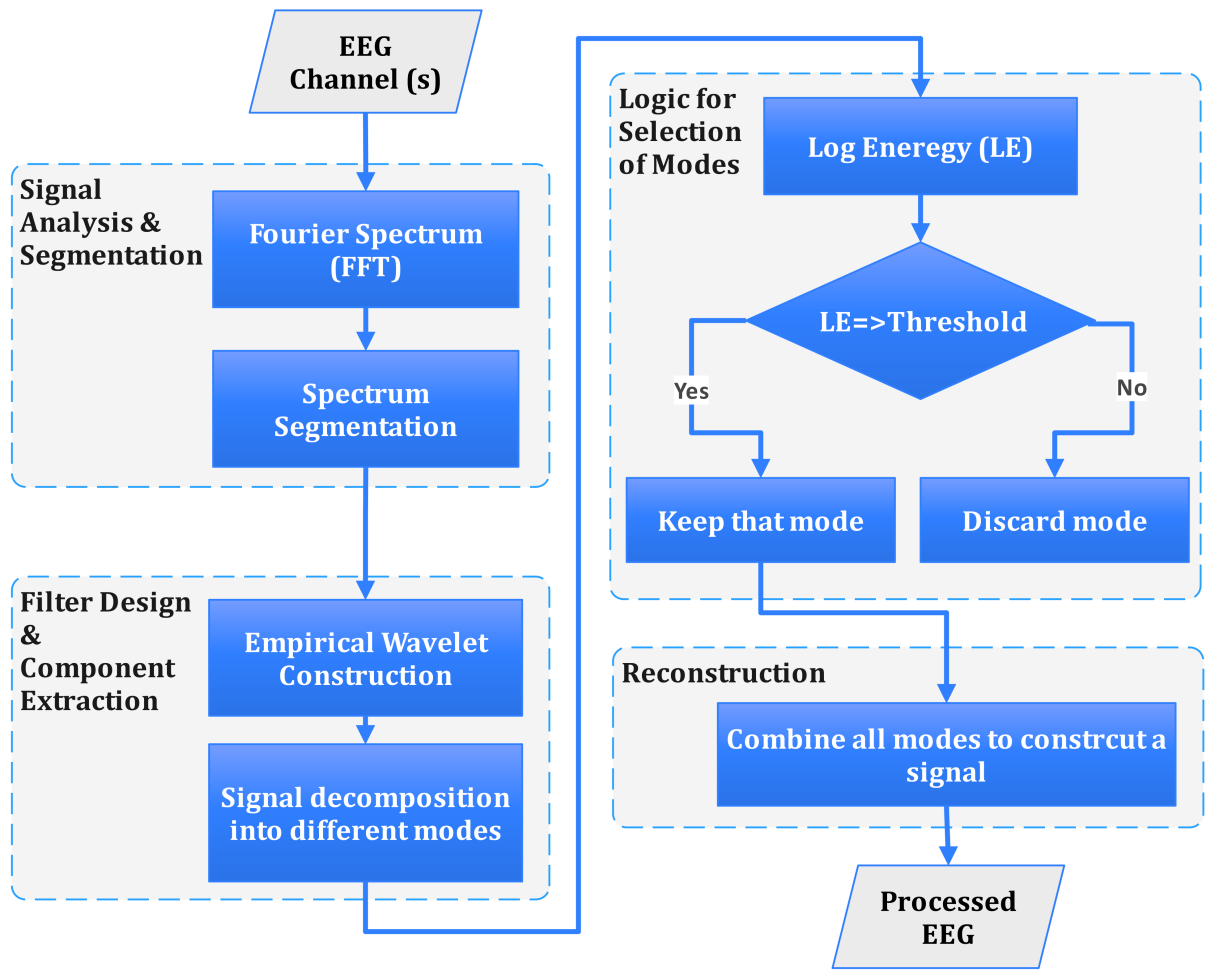
Steps involved in computing ALEEWR.

In response to these challenges, we introduce the Automated Log Energy-based Empirical Wavelet Reconstruction (ALEEWR). This novel approach leverages the strengths of EWT while incorporating an automated mechanism to enhance the selection of relevant modes based on their energy content. The Log operator incorporated in the ALEEWR plays a critical role in enhancing the detection of weak changes within EEG signals. The EEG channels were first normalised to remove any dependencies on gain. ALEEWR utilises a family of wavelets specifically adapted for particular signal processing applications. It extracts several modes from the Fourier transform of the input signal by creating adaptive wavelet filter banks. These modes are subsequently combined based on the log energy threshold to reconstruct a preprocessed signal. The significant steps of ALEEWR are presented in Figure 5 and are described next.

**Step 1:** Apply Fast Fourier Transform (FFT) to the input signal (s), where f(s) denotes its discrete version, $s=\{s_{i}\},i=1,2,3,\cdots ,n$. Here, *n* is the number of samples and the FFT spectrum denoted by X(w) is computed. Compute the set of maxima $n=\{n_{i}\},i=1,2,3,\cdots ,m$ of FFT spectrum and find their concerned frequencies $w=\{w_{i}\},i=1,2,\cdots ,m$. The number of maxima is denoted by *m*.

**Step 2:** The boundary detection algorithm is used to accurately separate the Fourier spectrum of the EEG signals. To find the border of each segment, the centre of two progressive local maxima is computed as defined by the following equation:

(5) $$\phi_{i}=\frac{W_{i}+W_{i+1}}{2}$$

where $\phi_{i}$ defines the boundaries set $\phi =\{\phi_{1},\phi_{1},\cdots ,\phi_{N-1}\}$ and $W_{i}$ and $W_{i+1}$ denotes two frequencies.

**Step 3:** In this stage, based on boundaries, an adaptive filter bank of *m* wavelet filter is designed. This adaptive filter consists of one low pass filter and m−1 bandpass filter. The following equation defines the relationships between boundaries and frequencies with the scaling function $\rho_{1}\left(w\right)$:

(6) $$\rho_{1}=\left\{\begin{array}{c} 1,|w|\leq \left(1-\sigma \right)\phi_{i}\\ cos\left(\frac{\pi }{2}\alpha \left(\sigma ,\phi_{1}\right)\right)\left(1-\sigma \right)\phi_{1}≺\left|w\right|\left(1+\sigma \right)\phi_{1}\\ 0,\;else \end{array}\right.$$

Empirical wavelets are denoted by $\Psi_{i}\left(w\right)$ and defined as follows:

(7) $$\Psi_{i}=\left\{\begin{array}{c} 1\left(1+\sigma \right)\phi_{i}≺\left|w\right|≺\left(1-\sigma \right)\phi_{i+1}\\ cos\left(\frac{\pi }{2}\alpha \left(\sigma ,\phi_{i+1}\right)\right)\left(1-\sigma \right)\phi_{i+1}\leq \left|w\right|\leq \left(1+\sigma \right)\phi_{i+1}\\ sin\left(\frac{\pi }{2}\alpha \left(\sigma ,\phi_{i}\right)\right)\left(1-\sigma \right)\phi_{i}\leq \left|w\right|\leq \left(1+\sigma \right)\phi_{i}\\ 0,\;else \end{array}\right.$$

where $\alpha \left(\gamma ,\phi_{i}\right)=\beta \left(\left(\frac{1}{2\gamma \phi_{i}}\right)\left(|w\right|-\left(1-\gamma \right)\phi_{i})\right)$. The γ function makes sure that there is no overlap between two successive transitions. The equation of γ is formed using the following relation:

(8) $$\gamma ≺min_{i}\left(\frac{\phi_{i+1}-\phi_{i}}{\phi_{i+1}+\phi_{i}}\right)$$

β(x) is a random function defined below as follows:

(9) $$\beta \left(x\right)=\left\{\begin{array}{c} 0x\leq 0\\ 1x\geq 1\\ \beta (x)+\beta (1-x)=1,x\in (0,1) \end{array}\right.$$

**Step 4:** Wavelet functions are applied to extract instantaneous frequency (IF) and instantaneous amplitude (IA) from each mode scaling. Approximate coefficients are the product of the scaling function with the input signal under consideration, defined as follows:

(10) $$W_{f}\left(0,s\right)=\langle f,\theta_{i}\rangle =\int f\left(\tau \right)\bar{\theta_{i}\left(\tau -s\right)d_{\tau }}$$

Similarly, detailed coefficients were obtained by multiplying input signal *f* by empirical wavelet as follows:

(11) $$W_{f}\left(i,s\right)=\langle f,\psi_{i}\rangle =\int f\left(\tau \right)\bar{\psi_{i}\left(\tau -s\right)d_{\tau }}$$

**Figure 6:**
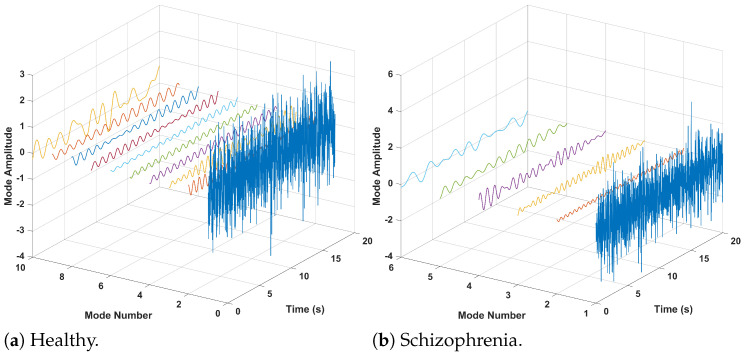
Multi-resolution analysis of EEG signal (1 channel) of healthy and schizophrenic subjects.

Here, $W_{f}\left(i,s\right)$ represents the detailed coefficients for the *i*th filter bank at the *s*th time instant. sub-band modes of both the healthy and schizophrenia classes are presented in Figure 6.

**Step 5:** To determine which sub-band modes contain the most information about subtle changes in the overall EEG signal, the log energy of each sub-band mode is calculated using the following equation:

(12) $$LE_{W_{f}}=\log\left(\sum_{n}{\left|W_{f}\right|}^{2}\right)$$

**Figure 7:**
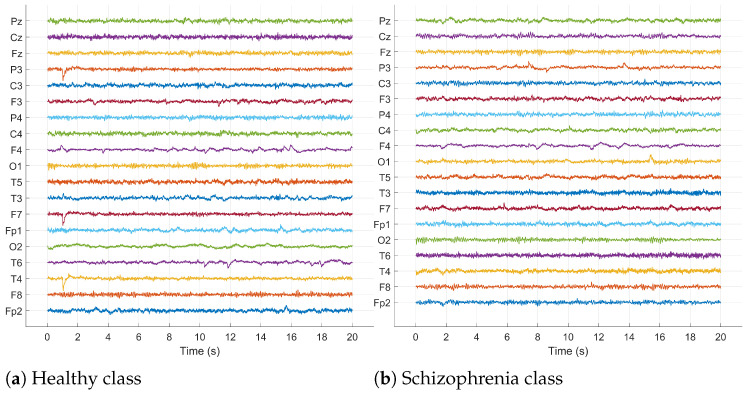
Preprocessed EEG signals of healthy and schizophrenic subjects using proposed ALEEWR.

In this equation, $LE_{W_{f}}$ represents the log energy of the sub-band. The modes that exhibit a log energy of 10% (a threshold experimentally selected) or higher are then combined to create a newly preprocessed EEG signal, effectively capturing and emphasising the subtle changes within the EEG data. The processed EEG channels after employing ALEEWR are shown in Figure 7. ALEEWR aims to optimise EEG signal preprocessing and feature extraction, providing a robust tool for handling weak and often noisy EEG signals. By automating the mode selection process, ALEEWR not only simplifies the preprocessing workflow but also enhances the reliability and accuracy of EEG signal analysis, thereby facilitating more precise diagnostics and research outcomes in neurological studies.

### 2.5. Feature Extraction: Novel Cepstral Features

To create reliable and relevant predictors, feature extraction is a crucial step in the learning process. Despite having sophisticated classification algorithms, low feature quality might result in poor performance and generalisation properties. In this study, we propose three powerful features for the detection of a schizophrenic EEG, i.e., cepstral activity, cepstral mobility, and cepstral complexity. These features provide numerical variations in the cepstrum of EEG channels, hence proving the fact that the EEG cepstrum contains rich schizophrenic information as compared to simple time-domain EEGs.

Real cepstrum *c* of input *x* can be defined as the inverse FFT of the logarithm FFT of EEG channels (Equation (13)).

(13) $$c_{x}=\frac{1}{2\pi }\int_{-\pi }^{\pi }log\left|X\left(e^{j\omega }\right)\right|e^{j\omega n}d\omega$$

In EEG signal analysis, capturing fine variations in both time and frequency domains is vital for accurately assessing brain activity, especially when differentiating between healthy and pathological conditions like schizophrenia. While conventional methods typically break down EEG signals into sub-bands such as alpha, beta, and gamma rhythms [39,40], this study extracts features from the entire EEG channels to account for individual differences in brain structure and function. Given the complexity of EEG signals and the issues with aliasing and overlapping frequencies, cepstral analysis provides a more comprehensive solution by isolating intrinsic periodicities and harmonics [41]. By extending beyond traditional time-domain or frequency-domain approaches, the cepstrum effectively decomposes the EEG signal into meaningful components, making it particularly suitable for non-stationary signals like EEG. We extracted three main features, cepstral activity, cepstral mobility, and cepstral complexity from the cepstrum of each EEG channel, which provide a detailed and refined characterisation of brain dynamics that traditional filters often fail to capture.

#### 2.5.1. Cepstral Activity

The cepstral activity (A) parameter quantifies the variance in the cepstrum of an EEG signal. It is analogous to the power of the signal in the cepstral domain and is defined as:

(14) $$A=\frac{1}{N}\sum_{i=1}^{N}{\left(c_{x}-\mu \right)}^{2}$$

where μ is the mean of the $c_{x}$, and *N* is the total number of cepstral coefficients.

#### 2.5.2. Cepstral Mobility

The cepstral mobility (M) parameter measures the rate of change in the cepstrum, analogous to the standard mobility parameter but applied to the cepstral coefficients of the EEG signal. It indicates the smoothness of the variations in the cepstrum and is defined as:

(15) $$M=\sqrt{\frac{A(d_{c})}{A(c_{x})}}$$

where $d_{c}$ is the derivative of the cepstral coefficients $c_{x}$, and $A(c_{x})$ and $A(d_{c})$ are the activities of the cepstral coefficients and their derivatives, respectively.

#### 2.5.3. Cepstral Complexity

Cepstral complexity (C) measures the irregularity or complexity of the cepstrum of the EEG. It evaluates how much the cepstral behaviour deviates from that of a simple sinusoidal form. This metric is defined as follows:

(16) $$C=\frac{M(d_{c})}{M(c_{x})}$$

where $M(c_{x})$ and $M(d_{c})$ are the mobilities of the cepstral coefficients and their derivatives, respectively.

These three Cepstral features were extracted from each of the 19 EEG channels, resulting in a comprehensive feature vector that captures the essential characteristics of the EEG signals across all channels. The feature vector, therefore, consists of 57 features.

### 2.6. Feature Reduction: ANOVA

The dimensions of the extracted EEG features were further reduced by eliminating irrelevant information or redundancy using a one-way analysis of variance (ANOVA). The objective of performing ANOVA is to determine whether different classes (or levels) of a factor have significantly different means. ANOVA evaluates the impact of each feature on the class label or response variable, identifying the features that significantly distinguish between classes [42,43].

Variation of the class is the overall mean, i.e., $\bar{v}.j-\bar{v}..$ (variation between classes), where v.j is the sample mean value of class *j* and $\bar{v}$ is the overall sample mean value. ANOVA examines the diversity in the class means by dividing the total variation in the feature data into two parts:

1. Variation of observations in each group from their group mean estimates;
2. Variation of instances in each class from their class means estimates $v_{ij}-\bar{v}.j$ (variation within a category).

ANOVA divides the total sum of squares (SST) into a sum of squares due to the between-classes effect (SSR) and the sum of squared error (SEE).

(17) $$\sum_{i}\sum_{j}{\left(v_{ij}-\bar{v}..\right)}^{2}=\sum_{j}n_{j}{\left(\bar{v}.j-\bar{y}\right)}^{2}+\sum_{i}\sum_{j}{\left(v_{ij}-\bar{v}.j\right)}^{2}$$

where $n_{j}$ is the sample size for the $j^{th}$ group, j=1,2,⋯,k.

ANOVA was used to identify the most significant features for classification as demonstrated in [44,45]. These features were then used to train and test the classification models. This process ensures that the selected features significantly contribute to distinguishing between the healthy and SZ classes.

### 2.7. Classification

The discrimination between the two classes of EEG signals was achieved through the application of various well-known machine learning classification algorithms. These models were decision trees (DTs), Support Vector Machines (SVMs), K-nearest neighbours (KNNs), ensemble classifiers, and Artificial Neural Networks (ANNs). The DT classifier predicts the output by following decisions in the tree structure, from the root node down to the leaf. The EEG signals of healthy controls and SZ subjects were also classified using a linear SVM classifier and its several non-linear kernel versions like Quadratic-SVM (QSVM) and Cubic-SVM (CSVM) [46]. Another classification method employed to differentiate healthy and SZ EEG signals using extracted features was KNN [47]. KNN works by finding the closest points to the new input [48]. The new input is assigned a class label based on the highest posterior probability of response of nearest neighbours. The ‘K’ value is the number of neighbours for voting. KNN with the value of K set to one is Fine KNN (FKNN) with Euclidean distance. KNN with the value of K set to 100 and squared inverse distance metric is Weighted KNN (WKNN). In Cubic KNN (CKNN), the cubic distance metric is used to measure the distance between current input features and dataset points.

The employment of ensemble machine learning algorithms for the classification of biomedical signals has received due attention [15,49]. In ensemble classification algorithms, the output is predicted using a set of learned classifiers in combination with some voting scheme. The resultant composite model is robust and often has better performance as compared to individual learners. Ensemble Boost Tree (EBoosTT) learns from the errors generated by a set of weak classifiers and turns them into a strong classifier using an iterative algorithm. AdaBoost with 30 decision tree learners was employed, and the number of splits was set to 20. The Ensemble Bagged Tree (EBagT) classification algorithm is constructed by bootstrapped training of several decision tree classifiers. The results of all predictors are averaged to produce the final output. The maximum number of splits was set to 1540 and the number of learners was 30. Ensemble subspace KNN (ESKNN) is assembled by combining several KNNs as base classifiers using a random subspace strategy [50]. The selected number of learners was 30 with a subspace dimension set to 40. Artificial Neural Networks (ANNs) are widely employed in biomedical signal processing for classification tasks [51,52]. An ANN contains neurons connected in input, hidden, and output layers. In this article, we analysed the classification performance using three types of ANN. An ANN structure with only one hidden layer is a Narrow Neural Network (NNN), with ten hidden layers (MNN), and with a hundred hidden layers is a Wide Neural Network (WNN). All networks used ReLu activations.

## 3. Results

The performance of the proposed EEG-based computer-aided diagnosis system for SZ was assessed using various well-known classification algorithms. The proposed features were extracted from all channels of EEG and were selected using ANOVA. Performance with KNN, DT, SVM, ANN, and Ensemble methods is presented. First, we present the feature importance analysis, then provide the classification performance of the best model, and finally, compare the performance of the proposed model with other classifiers. All experiments were conducted using 10-fold cross-validation to prevent overfitting, utilising MATLAB R2023b software on an Intel Core i7 system with 32 GB RAM. The dataset was divided into ten equal subsets, with each subset used for training while the remaining subsets were used for testing across ten iterations [9,53]. The experiments were repeated 20 times, and the average results were reported.

### 3.1. Feature Importance Determination

**Figure 8:**
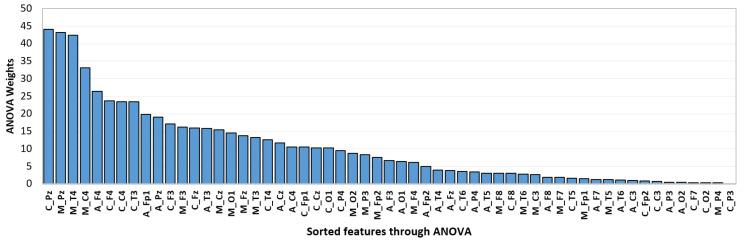
EEG feature ranking through ANOVA in a sorted manner.

Table 1 provides the results of applying ANOVA on all 57 features extracted from EEG signals. The importance of features is determined by the ANOVA weight. The higher value of weight signifies that the attribute is influential and contains strong discriminatory information. Figure 8 depicts the ANOVA-based weights for extracted EEG features in descending order. In Figure 8, $C_{Pz}$, $M_{Pz}$, and $A_{Pz}$ represent the proposed *cepstrum complexity*, *cepstrum mobility*, and *cepstrum activity* features extracted from the 19^*th*^ EEG channel (Pz), respectively. The classification was performed using ten features showing the highest weights ($C_{Pz}$, $M_{Pz}$, $M_{T4}$, $M_{C4}$, $A_{F4}$, $C_{F4}$, $C_{C4}$, $C_{T3}$, $A_{Fp1}$, $A_{Pz}$). It can be observed that features extracted from the 19^*th*^ EEG channel ($C_{Pz}$, $M_{Pz}$, $A_{Pz}$) have high ANOVA weights to be considered in the final feature vector. The statistical parameters in terms of mean, standard deviation (SD), and *p*-value of the principal features are shown in Table 2.

**Figure 9:**
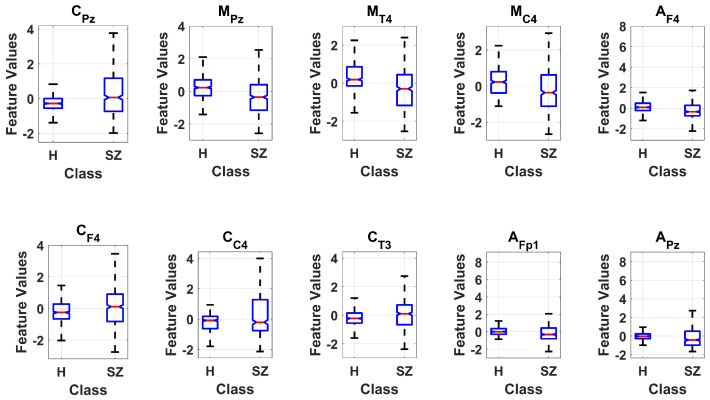
Box plots of the extracted features demonstrating the differences between healthy (H) and schizophrenia (SZ) classes.

Figure 9 illustrates the box plot analysis of the most significant features ranked using ANOVA, showing that the most discriminating information for healthy vs. SZ was available in the 19^*th*^ EEG channel. These ten most significant features were used to train and test the classification models using 10-fold cross-validation.

### 3.2. Performance of the Proposed Model

Table 3 enlists the experimental results of applying different versions of KNN for the classification of SZ and healthy EEG observations. The FKNN model achieved 99.4% accuracy, 99.21% sensitivity, and 99.6% specificity for distinguishing the healthy and SZ instances using only 10 features shown in Figure 9. A slightly low performance of 98.2% was obtained through WKNN, where the value of K is set to 100. This model attained 97.6% and 99% sensitivity and specificity, respectively. Comparatively poor performance of 69% accuracy was obtained using CKNN, where the cubic distance metric was employed instead of Euclidean. The overall best performance was obtained via FKNN, where out of 504 SZ observations, only 4 were misclassified as healthy. Similarly, only 2 out of 504 observations from healthy controls were misclassified by the model.

### 3.3. Performance Comparison with Other Classifiers

#### 3.3.1. Decision Tree

Table 4 provides detailed results of testing different variants of the decision tree classifier for distinguishing SZ and healthy EEG features. DTF is constructed using a large number of leaves and yields 93.1% classification accuracy. DTM contains a moderate number of branches and provides 91.3% prediction accuracy. A comparatively low accuracy of 74.4% was obtained using DTC, which uses a fewer number of splits. We observe a trend in reduction in accuracy as the number of branches in the decision tree goes down.

#### 3.3.2. Support Vector Machines

The experimental results of applying SVM with different kernel functions are shown in Table 5. LSVM was able to distinguish EEG features of healthy and SZ with 70.6% accuracy. Low performance confirms the complexity of the problem, as features of both classes were not linearly separable. QSVM provided enhanced results with accuracy reaching up to 95.1%, 92.5% sensitivity, and 97.8% specificity. The highest results of 96.5% accuracy were obtained through the CSVM. Non-linear kernel-based SVM classification models yielded better prediction performance as compared to linear SVM.

#### 3.3.3. Ensemble Classification Methods

Ensemble classifiers used a combination of various models with a voting strategy to predict the response. All ensemble classifiers used in this study have shown better performance, illustrated in Table 6. EBoosTT yielded an accuracy of 96.3% for the detection of SZ using extracted ten most significant cepstrum features. A slight increase in performance (96.9%) was shown using EbagT. Among all ensemble techniques, ESKNN achieved the highest results of 97.4% accuracy, 96.6% sensitivity, and 98% specificity.

#### 3.3.4. Artificial Neural Networks

Table 7 provides a performance analysis of applying different versions of ANNs for the classification of SZ and healthy EEG signals. Almost similar accuracy performances of 96.6% and 96.7% were observed using an NNN and WNN, respectively. These results demonstrated that raising the number of neurons in the hidden layer from 10 to 100 in narrow and wide networks has no substantial impact on the classification performance. A slightly better performance (97.1%) was obtained using MNN, which used 25 neurons in the hidden layers.

## 4. Discussion

**Figure 10:**
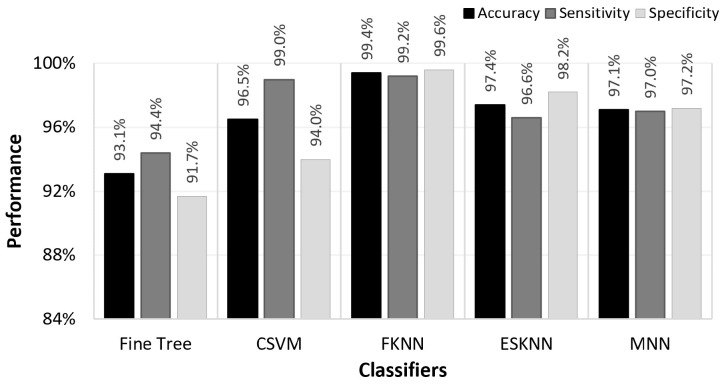
Comparison of the classification algorithms.

This research introduces a novel approach to the EEG-based diagnosis of schizophrenia, employing a combination of FastICA and ALEEWR for artefact removal, signal decomposition, and reconstruction. The proposed cepstral-based features further enhance diagnostic accuracy by capturing critical variations in the EEG signals. Through an ANOVA-based feature selection and classification via the FKNN algorithm, our system achieved an accuracy of 99.6%. Figure 10 shows the graphical comparison of performance in terms of accuracy, sensitivity, and specificity for several classifiers. The FKNN algorithm outperforms other classifiers with an accuracy of 99.4%, alongside high sensitivity (99.21%) and specificity (99.6%). The Fine Tree classifier shows a performance of 93%. The CSVM provides a high sensitivity of 99% but falls short of specificity compared to the FKNN. This indicates a slightly higher rate of false positives, which can be detrimental in clinical diagnostics. The ESKNN and MNN also demonstrate robust performance with accuracies above 97%, but they still do not reach the balanced performance of the FKNN in our specific application. The choice of the FKNN classifier is based not only on its superior accuracy but also on its ability to maintain balancing sensitivity and specificity, which is particularly advantageous in a clinical setting, where the accurate diagnosis of schizophrenia has profound implications for patient treatment and management.

**Figure 11:**
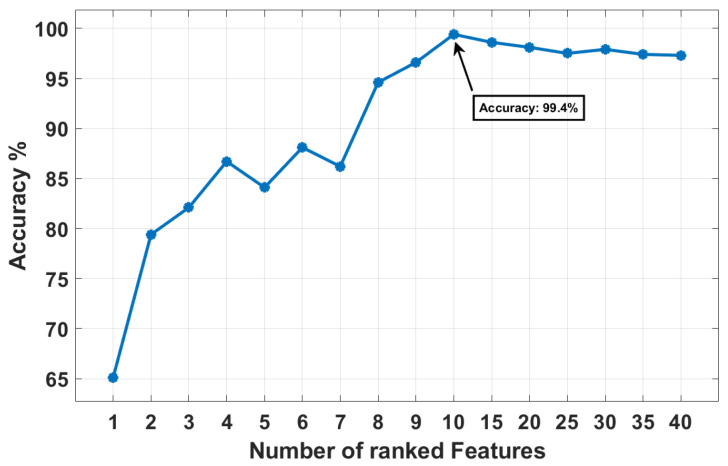
Accuracies of the proposed system across the ranked features.

Figure 11 illustrates the performance of the FKNN classifier with different numbers of features, showing a significant increase in accuracy as the number of features is incrementally increased from one to ten. Beyond ten features, the accuracy stabilises, peaking at 99.4% and maintaining a similar performance as additional features are considered up to forty. This trend indicates that a minimal set of ten features is sufficient for near-optimal diagnostic performance, as additional features do not significantly improve the classifier’s effectiveness, suggesting an optimal balance between feature complexity and diagnostic accuracy.

This performance significantly outperforms previous studies listed in Table 8. For instance, Zhang et al. [13] and Nikhil et al. [19] reported high accuracies of 98.5% and 99.0%, respectively, and utilised neural networks but did not focus on reducing feature space. Siuly et al. [15] used EMD for the classification of schizophrenia and healthy subjects with EEG signals, achieving an accuracy of 89.5%. However, their approach had the limitation of selecting an appropriate number of IMFs when using EMD. Jahmunah et al. [16] extracted 157 features from EEG signals and reduced them to 14 to achieve a 92.9% accuracy for the classification of schizophrenia and control groups. Kumar et al. [54] proposed a computer-aided approach for schizophrenia detection using EEG signals, employing local descriptors with a correlation-based feature selection algorithm. The reduced features are classified using AdaBoost, with temporal lobe EEG channels yielding the best performance of 99.3% accuracy. Das et al. [39] employed Multivariate Iterative Filtering with Hjorth parameters, achieving 98.9% accuracy using SVM. However, their approach required 30 features and involved extracting features from EEG bands, specifically delta, theta, alpha, beta, and gamma rhythms, which added significant computational overhead. The use of advanced EEG feature extraction techniques and KNN classification has proven effective. For instance, Akbari et al. [53] obtained an accuracy of 94.8% using 36 features from 12 channels using KNN classification. They used graphical features from the phase space dynamic of EEG signals. In another study, Aziz et al. [55] proposed brain textures for effective classification of schizophrenia using KNN classification and obtained 94.9% accuracy. The authors used EMD to decompose EEG into IMFs and after manual analysis, only the first two IMFs were added together to form a reconstructed preprocessed signal. Our method stands out by using fewer but highly discriminative features (10 features), with automated signal preprocessing through ALEEWR, eliminating the need for manual mode selection.

Deep learning methods, such as those employed by Lillo et al. [4], Wu et al. [21], and Oh et al. [17], typically require large datasets to train effectively. Lillo et al. employed a CNN and achieved an accuracy of 93%. Wu et al. utilised a Recurrent Auto-encoder, resulting in an accuracy of 81.8%. Oh et al. used a CNN and obtained a high accuracy of 98.0%. Hassan et al. [20] proposed a fusion of CNN and machine learning classifiers for schizophrenia classification, achieving 98% accuracy. They introduced a CNN-based channel selection mechanism, evaluating individual channels to assess their contribution to classification accuracy. However, a limitation of this approach is the need to select different channel subsets for each identification problem. The current algorithm lacks an automated channel selection method, making it highly dependent on the specific dataset used. Singh et al. [18] proposed a spectral analysis-based CNN model for identifying schizophrenia using multichannel EEG signals. The model processes EEG signals by filtering, segmenting, and converting them into the frequency domain, dividing them into six spectral bands: delta, theta-1, theta-2, alpha, beta, and gamma. However, CNN is computationally complex, and the extraction of spectral information from frequency bands further adds to the computational overhead, making the approach resource-intensive. The need for large datasets can be a significant challenge in medical contexts. Additionally, data augmentation techniques, which are less viable in medical contexts due to the high specificity required for accurate diagnosis, cannot always compensate for the lack of extensive data. Our proposed approach not only improves classification accuracy but also reduces computational complexity, making it feasible for continuous monitoring and embedded systems and edge computing. Moreover, the cepstral features introduced in our study offer a novel dimension of analysis not explored in other studies.

EEG signals are non-stationary and non-linear, with properties that change over time, making traditional time- and frequency-domain analyses insufficient. Cepstral analysis, transforming the signal into the quefrency domain, handles these complexities by analysing the power and rate of change in quefrency content. Additionally, cepstral features are less sensitive to noise and artefacts, which is crucial for reliable EEG analysis. This novel feature set, with its high discriminatory power, allows for precise differentiation between healthy and schizophrenic EEG signals, as evidenced by the high sensitivity (97.8%) and specificity (98.8%) achieved. In conclusion, our study advances the field of EEG-based schizophrenia diagnosis by introducing a method that not only improves diagnostic accuracy but also optimises computational efficiency and application potential in real-world settings. The introduction of ALEEWR and cepstral analysis as a methodological innovation represents a significant step forward in the automated diagnosis of schizophrenia, setting a new benchmark for future studies in this area.

## 5. Conclusions and Future Directions

In this study, we have presented an EEG-based framework for the early and precise diagnosis of SZ. The proposed framework employs Automated Log Energy-based Empirical Wavelet Reconstruction (ALEEWR) coupled with novel cepstral features. This multifaceted approach integrates advanced signal processing techniques with robust feature extraction and selection methods, significantly improving diagnostic accuracy and efficiency. By utilising FastICA for artefact removal and ALEEWR for signal reconstruction, we effectively enhance the EEG signal’s clarity and relevance. Subsequent extraction of cepstral features, i.e., cepstral activity, mobility, and complexity, provides a nuanced understanding of EEG dynamics, which is further refined through ANOVA-based feature selection. The employment of the Fine KNN classifier enables our system to achieve remarkable diagnostic performance with an accuracy of 99.40%, sensitivity of 99.21%, and specificity of 99.60%. These metrics not only underscore the effectiveness of our approach but also demonstrate its superiority over traditional diagnostic methods, which are often labour-intensive and prone to errors. Moving forward, the scalability of this framework offers promising avenues for broader clinical applications, ensuring robust, real-time diagnostics that can significantly impact patient outcomes and treatment strategies in mental health care.

However, there are some limitations to this study. Firstly, we used only one dataset to validate the algorithm. To ensure broader applicability and robustness, future studies should incorporate multiple datasets. Secondly, the data size is relatively small, which might limit the generalisability of our findings. Increasing the dataset size would help in better training and validation of the models. Thirdly, the current implementation of ALEEWR uses a static log energy threshold of 10%, which was selected experimentally. This threshold should be made adaptive to accommodate various signal-to-noise conditions dynamically.

Looking forward, this study sets the stage for significant advancements in schizophrenia diagnosis using EEG signals. Incorporating deep learning models like CNNs and LSTMs, particularly with cepstral features, promises deeper insights into EEG dynamics and potentially higher diagnostic accuracies. Expanding our dataset will further refine and validate our models, enhancing their generalisability and effectiveness. Additionally, integrating our methods into low-power, portable embedded systems could revolutionise mental health care delivery, enabling real-time, accessible diagnostics in remote settings. This approach not only aims to improve the clinical management of schizophrenia through earlier interventions but also optimises system design for energy efficiency and minimal computational demands, crucial for practical deployments in resource-limited environments.

## Figures and Tables

**Table 1:** Feature ranking results of applying ANOVA on extracted features from EEG signals.

Features	ANOVA Weights	Features	ANOVA Weights	Features	ANOVA Weights
$C_{Pz}$	44.1411	$A_{Cz}$	11.7543	$C_{F8}$	3.0421
$M_{Pz}$	43.2403	$A_{C4}$	10.5856	$M_{T6}$	2.7608
$M_{T4}$	42.4737	$C_{Fp1}$	10.4968	$M_{C3}$	2.6829
$M_{C4}$	33.1581	$C_{Cz}$	10.3155	$A_{F8}$	1.9436
$A_{F4}$	26.4594	$C_{O1}$	10.2426	$M_{F7}$	1.8861
$C_{F4}$	23.7429	$C_{P4}$	9.4774	$C_{T5}$	1.5994
$C_{C4}$	23.4761	$M_{O2}$	8.7708	$M_{Fp1}$	1.5252
$C_{T3}$	23.4276	$M_{P3}$	8.3576	$A_{F7}$	1.3270
$A_{Fp1}$	19.9070	$M_{Fp2}$	7.5335	$M_{T5}$	1.2159
$A_{Pz}$	19.1349	$A_{F3}$	6.6283	$A_{T6}$	1.1489
$C_{F3}$	17.1812	$A_{O1}$	6.4004	$A_{C3}$	0.9762
$M_{F3}$	16.2406	$M_{F4}$	6.1457	$C_{Fp2}$	0.8294
$C_{Fz}$	16.0346	$A_{Fp2}$	4.9863	$C_{C3}$	0.7992
$A_{T3}$	15.8375	$A_{T4}$	3.9301	$A_{P3}$	0.5133
$M_{Cz}$	15.5225	$A_{Fz}$	3.8874	$A_{O2}$	0.5116
$M_{O1}$	14.6075	$C_{T6}$	3.5882	$C_{F7}$	0.3653
$M_{Fz}$	13.7222	$A_{P4}$	3.5193	$C_{O2}$	0.3426
$M_{T3}$	13.3062	$A_{T5}$	3.0801	$M_{P4}$	0.3206
$C_{T4}$	12.6136	$M_{F8}$	3.0692	$C_{P3}$	0.1421

**Table 2:** Principal features selected using ANOVA.

Selected Features	Healthy		Schizophrenia		*p*-Value
	Mean	SD	Mean	SD	
$M_{Pz}$	0.79137	0.05777	0.74883	0.08591	$4.8\times 10^{-26}$
$M_{T4}$	0.79151	0.06131	0.7446	0.09756	$1\times 10^{-22}$
$M_{C4}$	0.80313	0.05694	0.76543	0.08949	$2\times 10^{-21}$
$C_{Pz}$	1.44425	0.12681	1.56597	0.26407	$4.8\times 10^{-20}$
$C_{C4}$	1.41478	0.1102	1.50065	0.27018	$1.6\times 10^{-12}$
$A_{F4}$	0.00211	0.00052	0.00183	0.00072	$2.4\times 10^{-11}$
$C_{T3}$	1.45274	0.12621	1.53644	0.2552	$2.1\times 10^{-10}$
$A_{Pz}$	0.00218	0.00063	0.00192	0.00077	$7\times 10^{-10}$
$C_{F4}$	1.47059	0.11914	1.54518	0.22191	$1\times 10^{-9}$
$A_{Fp1}$	0.00229	0.00068	0.002	0.00086	$7.5\times 10^{-9}$

**Table 3:** Performance of KNN classifiers using selected features for classification of normal and SZ EEG features.

Classifier	TN	FP	FN	TP	Acc	Sen	Sp	Prediction Speed	Training Time
FKNN	502	2	4	500	99.40%	99.21%	99.60%	19,000 obs/s	10.748 s
CKNN	499	5	301	203	69.60%	40.30%	99.00%	16,000 obs/s	1.039 s
WKNN	498	6	12	492	98.20%	97.60%	99.00%	20,000 obs/s	0.949 s

**Table 4:** Performance of DT using selected features for classification of normal and SZ EEG features.

Classifier	TN	FP	FN	TP	Acc	Sen	Sp	Prediction Speed	Training Time
Fine Tree	462	42	28	476	93.10%	94.40%	91.70%	5000 obs/s	10.328 s
Medium Tree	472	32	56	448	91.30%	88.90%	93.70%	10,000 obs/s	5.411 s
Coarse Tree	393	111	147	357	74.40%	70.80%	78.00%	11,000 obs/s	3.178 s

**Table 5:** Performance of SVM using selected features for classification of normal and SZ EEG features.

Classifier	TN	FP	FN	TP	Acc	Sen	Sp	Prediction Speed	Training Time
LSVM	437	67	229	275	70.60%	54.60%	86.70%	9500 obs/s	80.473 s
QSVM	493	11	38	466	95.10%	92.50%	97.80%	12,000 obs/s	7.1329 s
CSVM	474	30	5	499	96.50%	99.00%	94.00%	12,000 obs/s	4.833 s

**Table 6:** Performance of Ensemble classifiers using selected features for classification of normal and SZ EEG features.

Classifier	TN	FP	FN	TP	Acc	Sen	Sp	Prediction Speed	Training Time
EBoosTT	493	11	26	478	96.30%	94.80%	97.80%	5700 obs/s	9.667 s
EBagT	494	10	21	483	96.90%	95.80%	98.00%	6400 obs/s	4.677 s
ESKNN	495	9	17	487	97.40%	96.60%	98.00%	2800 obs/s	3.541 s

**Table 7:** Performance of ANNs using selected features for classification of normal and SZ EEG features.

Classifier	TN	FP	FN	TP	Acc	Sen	Sp	Prediction Speed	Training Time
NNN	491	13	21	483	96.60%	95.80%	97.40%	31,000 obs/s	15.206 s
MNN	490	14	15	489	97.10%	97.00%	97.20%	44,000 obs/s	1.748 s
WNN	487	17	16	488	96.70%	96.80%	97.00%	39,000 obs/s	1.522 s

**Table 8:** Summary of EEG-based studies on schizophrenia detection.

Study	Method	Results
[12]	Event-related potential features, Random Forest	81.1%
[22]	Multi-domain connectome CNN	91.7%
[23]	Deep Belief Network	95.0%
[16]	Non-linear features, *t*-test	92.9%
[17]	Convolutional Neural Network	98.0%
[11]	Graph Theory-based Network Connectivity Analysis	82.3%
[13]	Artificial Neural Network	98.5%
[15]	Empirical Mode Decomposition, Ensemble Bagged Tree	89.5%
[19]	Long short-term memory	99.0%
[9]	Logistic Regression	97.0%
[53]	Graphical Features, KNN	94.8%
[39]	Multivariate Iterative Filtering, Hjorth parameters	94.8%
[18]	Spectral Features, CNN	98.5%
[4]	Convolutional Neural Network	93.0%
[21]	Recurrent Auto-encoder	81.8%
[55]	Brain Textures, KNN	94.9%
[20]	Convolutional Neural Network, Logistic Regression	98.0%
[7]	Spectrogram, Local Configuration Patterns	97.2%
[54]	Local descriptors, AdaBoostM1	99.3%
This work	FastICA, ALEEWR, Cepstral Features, FKNN	99.4%

## Data Availability

For the development and validation of the proposed method, we used a public domain dataset [24].
